# Subgingival Plaque Removal Efficacy and Oral Soft Tissue Safety of the Wave Electric Toothbrush: An In Vitro and In Vivo Study

**DOI:** 10.3390/dj14010029

**Published:** 2026-01-04

**Authors:** Siyuan Huang, Weidong Du, Jie Wu, Yunyang Lu, Weili Ku, Xiliu Zhang, Dongsheng Yu

**Affiliations:** Guangdong Provincial Key Laboratory of Stomatology, Guanghua School of Stomatology, Hospital of Stomatology, Sun Yat-sen University, Guangzhou 510055, China; huangsiyuansarah@163.com (S.H.); duwd@mail2.sysu.edu.cn (W.D.); wujie68@mail2.sysu.edu.cn (J.W.); luyy27@mail2.sysu.edu.cn (Y.L.); kuwli@mail2.sysu.edu.cn (W.K.); zhangxliu3@mail.sysu.edu.cn (X.Z.)

**Keywords:** oral hygiene, electric toothbrush, gingival sulcus cleaning, soft tissue safety, dentistry, periodontology

## Abstract

**Background/Objectives**: The novel wave electric toothbrush is considered potentially helpful in removing subgingival plaque to prevent the occurrence of periodontal diseases. This study aimed to assess the cleaning efficacy of a novel wave electric toothbrush on subgingival plaque and its safety profile for oral soft tissues. **Methods**: In vitro cleaning efficacy evaluations were conducted using oral dental models. The wave electric toothbrushes were divided into low-, medium-, and high-swing parameter groups, with manual brushing (Bass technique) as the control. Simulated plaque was applied to the buccal and gingival sulcus sites of the four first molars, and the plaque removal area and sulcus cleaning depth were measured. For safety evaluation, Sprague Dawley (SD) rats were brushed on their molars daily for 30 days, with bleeding incidents recorded. Oral soft tissues were analyzed through H&E staining and immunohistochemical analysis. Statistical analysis included ANOVA and Kruskal–Wallis (*p* < 0.05). **Results**: Medium- and high-swing groups demonstrated superior gingival sulcus cleaning efficacy, showing significant differences compared with the low-swing and control groups (*p* < 0.05). All swing parameters achieved complete plaque removal on buccal surfaces. No significant differences were observed between the low-swing and manual groups, or between the medium- and high-swing groups, regarding sulcus cleaning efficacy and maximum sulcus depth. During the 30-day in vivo experiment, medium- and high-swing groups showed low bleeding frequencies, primarily at the palatal gingiva and vestibule. Histological analyses indicated that higher swing parameters increased the likelihood of soft tissue injury. **Conclusions**: Wave electric toothbrushes enhance subgingival plaque removal, with higher swing parameters improving gingival sulcus cleaning. However, stronger parameters may increase the risk of soft tissue damage. Further clinical studies are required to establish optimal guidelines.

## 1. Introduction

Approximately 3.5 billion people worldwide are affected by oral diseases such as dental caries and periodontal diseases, primarily due to poor oral hygiene, especially the accumulation of plaque in the gingival sulcus [1,2,3]. Despite recommendations for individuals to brush their teeth twice a day using the Bass technique, the effective prevention of oral diseases through correct and regular toothbrushing has been well established [4]. Correctly using the Bass technique for cleaning tooth surfaces and the gingival sulcus appears to be extremely challenging, as individuals often struggle to master the technique of positioning the bristles at 45° towards the gingival margin, allowing the bristles to enter the gingival sulcus and perform small horizontal vibrations to remove subgingival plaque, while patiently cleaning each tooth [5]. With the growing demand for oral health and effective tooth cleaning, various cleaning techniques have developed, leading to the widespread adoption of electric toothbrushes [6,7,8]. Electric toothbrushes not only demonstrate higher efficiency in plaque removal and maintaining gingival health, but also offer advantages such as ease of use and improved patient compliance [9,10]. In addition, studies have shown that electric toothbrushes also have positive effects in orthodontic treatment, restorative care, and periodontal therapy [11,12,13,14,15,16]. This category of toothbrushes is divided into rotating and vibrating types based on the motion of the brush head [17].

Both rotating and vibrating electric toothbrushes are powered by electricity to drive the brush head in high-frequency reciprocating movements. The bristles on the brush head use friction to directly act on the tooth surface, removing dirt and plaque. For gingival sulcus cleaning, rotating electric toothbrushes primarily rely on the direct friction of the bristle tips to remove plaque at the gingival margin and near the gingival sulcus. In contrast, vibrating electric toothbrushes generate high-speed water flow and microbubbles in the oral cavity through the high-frequency movement of the bristles, which impact the gingival sulcus [18,19]. However, the bristle movement of these two types of electric toothbrushes typically only cleans the gingival sulcus to a depth of 0.5–1 mm [20], whereas plaque in healthy individuals generally accumulates subgingivally in the 0–3 mm range [21]. Studies have shown that if plaque in the 0–3 mm range of the gingival sulcus is not effectively cleaned over the long term, it can lead to gingivitis and other diseases [22]. Therefore, altering the motion of the electric toothbrush brush head to allow the bristles to enter 0–3 mm subgingivally and directly friction-clean the gingival sulcus is a potentially effective method for cleaning the gingival sulcus. This approach could enable comprehensive plaque removal from the oral cavity, thereby effectively preventing oral diseases.

Recently, a new wave electric toothbrush has emerged and rapidly gained market share in China, with representative brands like Laifen and Usmile being prominent drivers of this growth. Building on the vibrating electric toothbrush, it uses a servo motor to achieve high-frequency lateral oscillation of the brush head. The brush head’s maximum oscillation angle can reach 60 degrees, simulating the sweeping motion of the Bass technique, allowing the bristles to penetrate deeply into the gingival sulcus for cleaning. Therefore, it may offer superior gingival sulcus and oral cleaning efficacy. However, the high-frequency, large-angle oscillation of the bristles increases the likelihood of the bristles coming into contact with the oral soft tissues while cleaning the gingival sulcus. Therefore, it is essential to evaluate its effectiveness in cleaning the gingival sulcus and its soft tissue safety to ensure the oral health of the user. Currently, research on the cleaning efficacy and safety of wave electric toothbrushes remains limited.

Therefore, this study aims to investigate the oral cleaning efficacy and soft tissue safety of the wave electric toothbrush by constructing oral dental models and using SD rats as experimental subjects. The factors that may interfere with oral cleaning efficacy and soft tissue safety include the swing parameters of the wave electric toothbrush brush head. The null hypotheses are as follows: (1) There is no difference in oral cleaning efficacy between the wave electric toothbrush and traditional manual brushing; (2) the swing parameters of the wave electric toothbrush brush head do not affect cleaning efficacy; (3) there is no difference in soft tissue safety between the wave electric toothbrush and traditional manual brushing.

## 2. Materials and Methods

### 2.1. Utilization of Typodont Teeth and Animal Research Ethics

In this study, typodont teeth made of resin material were firmly mounted on a standardized dental model to construct an in vitro oral model (Alpha Dental Equipment Co., Ltd., Guangzhou, China) [16,23,24,25]. Additionally, the animal experiments involved in this study were approved by the Animal Ethics Committee of Sun Yat-sen University, adhering to the principles of animal protection, welfare, and ethics. The ethics approval number is SYSU-IACUC-2024-001264.

### 2.2. In Vitro Gingival Sulcus Cleaning Efficacy Experimental Design

**Figure 1:**
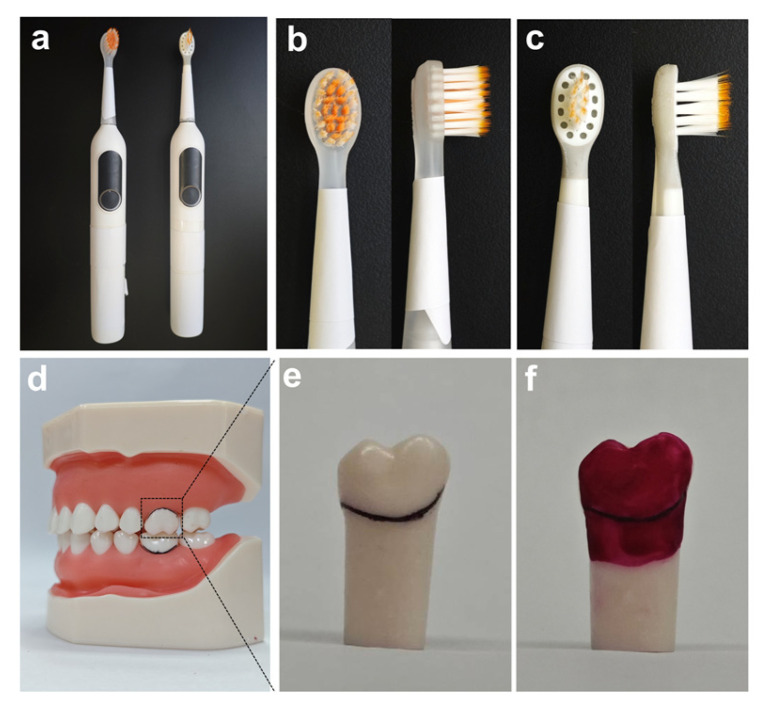
(**a**) Photo of the wave electric toothbrush. (**b**) Brush head used for the in vitro experiment. (**c**) Brush head used for the in vivo experiment. (**d**) In vitro oral cleaning model. (**e**) Experimental test tooth. (**f**) Experimental test tooth coated with simulated plaque on the buccal surface and gingival sulcus area.

The gingival sulcus cleaning efficacy in the oral model was the primary outcome measure, while the cleaning efficacy of the tooth surface was the secondary outcome measure. This study primarily evaluated the cleaning efficacy of wave electric toothbrushes (Starpulse Co., Ltd., Guangzhou, China) with high (brush head swing angle: 22°), medium (14°), and low (9°) swing parameters (Figure 1a). In addition, a manual brushing group using the Bass technique and a blank control group were set up for comparison. The brushing time, simulated plaque coating conditions, and cleaning positions were standardized. After brushing, measurements and calculations were made to obtain the buccal surface cleaning area, maximum gingival sulcus cleaning depth, and gingival sulcus cleaning area.

In the oral cleaning experiment, simulated dental plaque was prepared with modifications based on previous methods [26,27]. The plaque was prepared by mixing cornstarch (Aladdin, Shanghai, China), water (Watsons, Guangzhou, China), fuchsin (Aladdin, Shanghai, China), glycerin (Aladdin, Shanghai, China), and ethanol (Aladdin, Shanghai, China) in a mass ratio of 50:33:1:19:12. Then, the simulated plaque was evenly applied to the buccal surface and gingival sulcus of the upper right first molar (16), upper left first molar (26), lower left first molar (36), and lower right first molar (46), as shown in Figure 1d,f. Moreover, a black line was used to distinguish the buccal surface from the gingival sulcus (Figure 1e). Subsequently, the oral model with uniformly applied simulated plaque was transferred to an oven (Blue Pard, Shanghai, China) and maintained at 60 °C for 30 min to allow solvent evaporation, ensuring firm adherence of the plaque. Subsequently, the four first molars were reinserted into the dental model for the tooth brushing experiment.

### 2.3. Cleaning Performance Evaluation

The cleaning efficacy of the wave electric toothbrush on the oral model was assessed by simulating daily toothbrushing, brushing the entire oral model with 0.2 g of toothpaste (Colgate-Palmolive, Guangzhou, China) each time. Prior to conducting the in vitro oral cleaning experiment, the operators received systematic training focused on mastering standardized brushing sequences and contact pressure. To simulate daily human brushing, a strict brushing sequence was followed: starting from the upper left quadrant, then proceeding sequentially to the upper right, lower right, and finally the lower left quadrant, with each quadrant allocated 30 s, resulting in a total duration of 2 min. Using a thin-film pressure sensor (RunesKee, Shenzhen, China), the operator was trained to maintain a consistent pressure of approximately 1.5 N on each tooth during the simulated brushing process [28]. After brushing, the right upper first molar (16), left upper first molar (26), left lower first molar (36), and right lower first molar (46) were selected as representative samples for cleaning efficacy evaluation. In this experiment, the wave electric toothbrush head was equipped with a full set of bristles, as shown in Figure 1a,b.

To evaluate the cleaning efficacy of the wave electric toothbrush on the buccal surface and buccal gingival sulcus, photographs were taken with equal distance and magnification. The dental model was placed in a fixed position, with the camera (Canon, Oita, Japan) position and parameters also fixed. A ruler was placed vertically at a fixed location as a scale. Then, the images were then imported into ImageJ software version 1.54g (National Institutes of Health, Bethesda, MD, USA) to calculate the buccal surface cleaning area (S_buccal_), maximum gingival sulcus cleaning depth (L), and gingival sulcus cleaning area (S_sulcus_). The area was calculated using the following formula [29]:

S_actual_ = S_pixels_ × (L_actual_/L_pixels_) (1)

where S_actual_ represents the actual area of the target region, S_pixels_ is the pixel area of the target region, L_actual_ is the actual length of the scale, and L_pixels_ is the pixel length of the scale in the image. In addition, the entire experimental procedure was repeated six times to minimize experimental error.

### 2.4. In Vivo Safety Experiment Design Using Animal Models

The in vivo animal experiment randomly assigned SD rats (Beijing Vital River Company, Beijing, China) into 5 groups: wave electric toothbrush with high swing parameters, wave electric toothbrush with medium swing parameters, wave electric toothbrush with low swing parameters, manual brushing group, and blank control group. Over a 30-day period, the rats were brushed once daily, and the number of bleeding episodes during brushing was recorded [30].

The selection of SD rats was based on the criteria used in previous studies [31,32], and the inclusion criteria were as follows: male SD rats aged 10–12 weeks, weighing 240–300 g, with no systemic diseases, normal oral structure and function, ability to eat and drink normally, and normal behavioral activity. The exclusion criteria were as follows: presence of systemic diseases or oral diseases, use of medications or treatments within the last 6 months that could affect the oral cavity, oral injuries caused by fighting or self-mutilation, and the presence of oral developmental abnormalities.

### 2.5. Soft Tissue Safety Assessment

The safety evaluation of the wave electric toothbrush on oral soft tissues was based on the incidence of soft tissue bleeding and tissue damage in SD rats. To study the impact of different brush head swing parameters on oral soft tissues, 35 SD rats were randomly assigned into 5 groups, with 7 rats per group. Trained experimental staff used the wave electric toothbrush to brush all anesthetized rats, ensuring that the brush head’s vibration angle (14.4°) and vibration frequency (190 Hz) remained consistent, with only the swing parameters being adjusted. Each brushing session used 0.1 g of toothpaste, with the first to third molars in the upper molar region of the rats selected as the primary brushing sites. The total brushing time was standardized to 1 min, with 30 s allocated to the left and right sides. Bleeding was observed and recorded after each session. In this experiment, the brush head of the wave electric toothbrush had a partial bristle configuration compared to a full brush head, as shown in Figure 1a,c. After 30 days of brushing, the rats were euthanized, and soft tissue samples were collected from different oral sites for H&E staining and immunohistochemical analysis (TNF-α and IL-1β). For immunohistochemical quantification, a blinded assessment was conducted following a standard protocol [33]. All reagents used in this experiment were supplied by Servicebio Technology Co., Ltd. (Wuhan, China). The average expression level per animal (*n* = 7 biological replicates) was used for statistical comparison. The samples were observed under a microscope, and images were captured. H&E staining results were used to directly observe cellular and tissue structures, while ImageJ software was employed to quantify the localization and staining intensity of TNF-α and IL-1β proteins in the oral soft tissues of the SD rats.

### 2.6. Statistical Analysis

Statistical analyses were performed using SPSS version 30.0 (SPSS Inc., Chicago, IL, USA). Normality and homogeneity of variances were assessed with the Shapiro–Wilk and Levene’s tests, respectively. Data from the in vitro cleaning assay and oral soft tissue bleeding counts violated parametric assumptions (*p* < 0.05); thus, non-parametric tests were used. Group differences were analyzed with the Kruskal–Wallis test, followed by Dunn’s post hoc test with Bonferroni correction for pairwise comparisons. In contrast, immunohistochemistry data met parametric assumptions (*p* > 0.05) and were analyzed by one-way ANOVA, with significant results further tested using Tukey’s HSD post hoc test. The significance threshold was set at *p* < 0.05. In figures, significance is denoted as ns (not significant), * *p* < 0.05, ** *p*< 0.01, *** *p* < 0.001.

## 3. Results

### 3.1. Cleaning Efficacy of Buccal Surface and Gingival Sulcus

**Figure 2:**
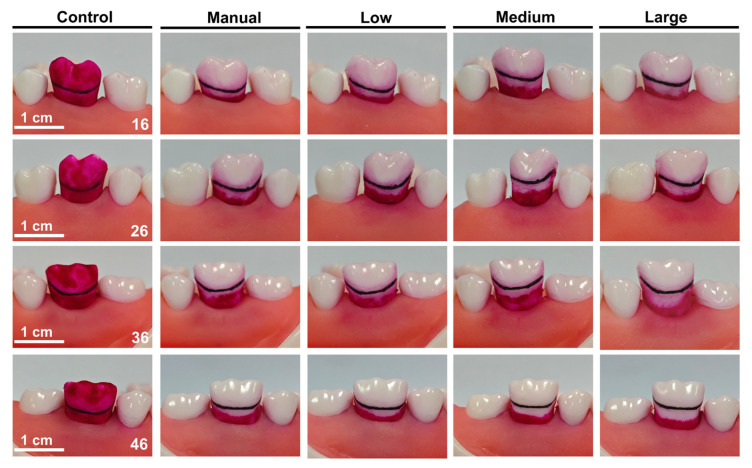
In vitro cleaning experiment images showing plaque removal from different tooth positions.

The evaluation of the wave electric toothbrush’s cleaning efficacy was conducted by simulating the daily toothbrushing process and brushing the entire oral model. The buccal surface and gingival sulcus of the upper right first molar (16), upper left first molar (26), lower left first molar (36), and lower right first molar (46) were selected as the areas for plaque cleaning efficacy testing. The boundary between the buccal surface and gingival sulcus was marked in black to facilitate observation and calculation. The plaque-covered areas on the buccal surfaces of teeth 16, 26, 36, and 46 were 62.45, 61.08, 69.94, and 70.09 mm^2^, respectively. The area beneath the gingival margin up to 3 mm was defined as the gingival sulcus region, with gingival sulcus areas on teeth 16, 26, 36, and 46 measuring 24.88, 25.64, 27.48, and 27.60 mm^2^, respectively. The cleaning efficacy results of the wave electric toothbrush are shown in Figure 2 and Table 1.

In the evaluation of gingival sulcus plaque cleaning efficacy, significant differences were observed between the wave electric toothbrush with medium and high swing parameters and the manual Bass brushing group (*p* < 0.05). Compared to the manual group, low swing parameter group, and medium swing parameter group, the wave electric toothbrush with high swing parameters demonstrated the highest gingival sulcus plaque removal rate and maximum gingival sulcus cleaning depth. Both the manual group and the wave electric toothbrush group were able to completely remove plaque from the buccal surface. No significant differences were observed between the manual group and the low swing parameter group in terms of gingival sulcus cleaning efficacy (*p* > 0.05) and maximum gingival sulcus cleaning depth (*p* > 0.05). Similarly, no significant differences were found between the medium and high swing parameter groups in terms of gingival sulcus cleaning efficacy (*p* > 0.05) and maximum gingival sulcus cleaning depth (*p* > 0.05). Furthermore, the wave electric toothbrush with low swing parameters showed a significant difference in gingival sulcus cleaning area compared to the medium and high swing parameter groups (*p* < 0.05). In terms of maximum gingival sulcus cleaning depth, significant differences were observed between the low and high swing parameter groups (*p* < 0.05), but no significant difference was found between the low and medium swing parameter groups (*p* > 0.05).

### 3.2. Oral Bleeding Conditions

The occurrence of oral bleeding in SD rats under different working parameters of the wave electric toothbrush is shown in Table 2. During the 30-day experiment, no oral bleeding was observed in the control and manual groups. As the brush head swing parameters of the wave electric toothbrush increased, the number of bleeding episodes in the SD rats’ oral cavity rose from 0 in the low parameter group to 1.17 ± 1.1 in the medium parameter group, and 3.33 ± 2.8 in the high parameter group. Comparison of bleeding conditions between different brush head swing parameters showed significant differences between the low parameter group and the medium parameter group (*p* < 0.05), as well as between the low parameter group and the high parameter group (*p* < 0.001).

**Figure 3:**
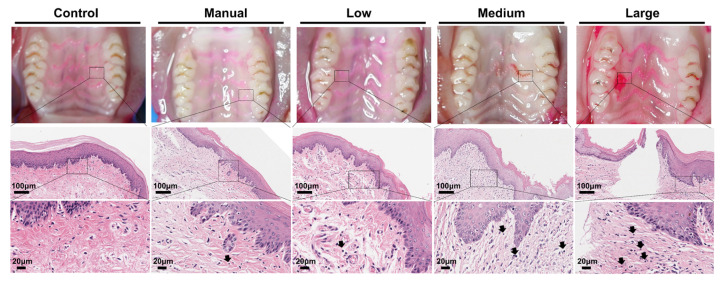
Photographs of the oral upper jaw of rats and H&E staining of rat oral soft tissues (magnification 20× and 60×, black arrows indicating lymphocytes).

Furthermore, observations of the upper jaw in different groups of SD rats during tooth brushing (Figure 3) revealed that bleeding in the oral soft tissues primarily occurred at the palatal gingiva and within the oral vestibule.

### 3.3. H&E Staining

H&E staining revealed that in the unbrushed control group, the epithelial layer structure of the oral soft tissues was intact and well-organized, with uniform distribution of chromatin in the cell nuclei and no abnormal changes. No infiltration of inflammatory cells such as lymphocytes or neutrophils was observed in the connective tissue, and the tissue morphology appeared normal (Figure 3). In the manual brushing group, the epithelial layer of the soft tissues showed localized thinning, with slight disorganization of the cell arrangement. A small number of lymphocytes were present in the connective tissue, indicating mild soft tissue damage caused by manual brushing. Compared to the control and manual groups, the use of the wave electric toothbrush resulted in increased soft tissue damage as the swing parameters of the brush head were enhanced. After 30 days of continuous brushing with the low swing parameter wave electric toothbrush, there was a reduction in the number of upper epithelial layers, and the extent of inflammatory cell infiltration in the connective tissue increased, with the damage level being similar to that of the manual brushing group. The medium swing parameter group exhibited increased disruption of the epithelial layer structure, with further infiltration of inflammatory cells. In the high swing parameter group, large areas of epithelial layer loss were observed, directly exposing the underlying connective tissue. Inflammatory cell infiltration was dense, and fibroblast proliferation was also visible.

### 3.4. Immunohistochemical Analysis

**Figure 4:**
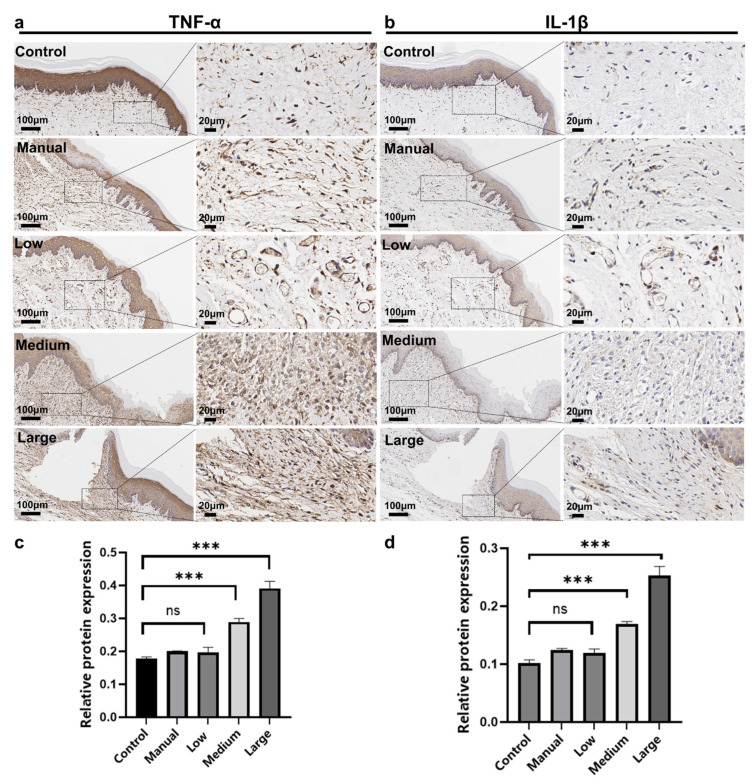
The images of immunohistochemical staining of (**a**) TNF-α, and (**b**) IL-1β. The quantitative analysis of (**c**) TNF-α, and (**d**) IL-1β. Data were presented as mean ± SD, and significant differences were determined by one-way ANOVA followed by Tukey’s HSD post hoc test. (*n* = 7, *** *p* < 0.001, ns: no significance).

The immunohistochemical staining for TNF-α showed that in the control group, the oral soft tissue exhibited normal structure with very low baseline expression of TNF-α (Figure 4a). In the manual brushing group, stromal cells in the connective tissue displayed brown-yellow staining, and TNF-α expression in the low swing parameter group was similar to that in the manual brushing group. In the medium and high swing parameter groups, TNF-α positive signals were widely distributed in the connective tissue, with the highest TNF-α expression observed in the high swing parameter group after using the wave electric toothbrush. Quantitative analysis indicated that, compared with the control group, TNF-α expression levels increased significantly by 51.4% and 104.8% in the medium and high swing parameter groups, respectively (*p* < 0.001; Figure 4c). Regarding the expression of the key pro-inflammatory cytokine TNF-α in SD rat oral soft tissue cells, no significant differences were detected between the control, manual, and low swing parameter groups. However, significant differences were observed between the low swing parameter group and the medium swing parameter group (*p* < 0.001), as well as between the low swing parameter group and the high swing parameter group (*p* < 0.001). The immunohistochemical staining results for IL-1β (Figure 4b) were highly consistent with those for TNF-α. Specifically, IL-1β expression levels increased by 61.4% and 141.8% in the medium and high swing parameter groups, respectively, compared with the control group. These findings further suggest that stronger brush head swing parameters of the wave electric toothbrush are more likely to trigger an inflammatory response.

## 4. Discussion

The findings of this study provide valuable insights into the oral cleaning efficacy of the wave electric toothbrush under in vitro conditions, demonstrating its effective cleaning on both the gingival sulcus and the buccal surface of the teeth. The results indicate that the cleaning efficacy of the wave electric toothbrush with medium and high swing parameters differed significantly from that of manual brushing, particularly in the cleaning of the gingival sulcus. Therefore, the first null hypothesis was rejected.

The observed improvement in oral cleaning efficacy may be closely related to the brush head movement pattern of the wave electric toothbrush. We used the dental model to simulate the daily toothbrushing process, with the Bass technique serving as the positive control in this experiment [34,35]. This is because, among the various brushing methods developed, such as the Bass technique, rolling method, and horizontal brushing method, only the Bass technique is effective in cleaning plaque from the gingival sulcus [36,37]. More critically, if plaque in the gingival sulcus is not effectively removed over time, bacteria within the sulcus (such as *Porphyromonas gingivalis*, *Tannerella forsythia*, and *Treponema denticola*) and their metabolic toxins can stimulate the gingival tissue, leading to an inflammatory response, manifesting as redness, swelling, bleeding, and bad breath. If left untreated, this inflammation may spread deeper, progressing to irreversible periodontitis [38,39]. Additionally, it has been linked to various chronic human diseases, including inflammatory bowel disease, cancer, cardiovascular diseases, Alzheimer’s disease, diabetes, rheumatoid arthritis, and preterm birth [40]. Therefore, effective cleaning of the gingival sulcus during daily tooth brushing is crucial. However, correctly performing the Bass technique is difficult for most individuals, as manual brushing often involves improper execution of the Bass technique and inadequate brushing duration [41]. Therefore, the wave electric toothbrush simulates the Bass brushing technique by using a servo motor to control the high-frequency lateral oscillation of the brush head while strictly controlling the brushing time, thus achieving effective oral cleaning. During the brushing process, the brush head oscillates laterally, causing the bristles to move back and forth. As the brush moves across the boundary between the tooth surface and the gingival sulcus, the reciprocating bristles continuously enter the gingival sulcus and provide friction, thereby cleaning the sulcus.

The research results indicate that wave electric toothbrushes with different swing parameters are capable of completely removing plaque from the buccal surface, demonstrating their effective cleaning ability. Furthermore, for the more challenging gingival sulcus cleaning, the Bass technique can remove a portion of the plaque in the gingival sulcus through the bristles’ sweeping motion as they enter the sulcus [42]. The in vitro cleaning experiment showed that when the wave electric toothbrush operated with low swing parameters, the gingival sulcus cleaning efficacy and maximum gingival sulcus cleaning depth were comparable to those of the Bass technique. The results above indicate that the wave electric toothbrush, through the reciprocating oscillation of the bristles at the junction of the gingival sulcus and tooth surface, allows the bristles to enter the gingival sulcus and effectively clean the plaque within the sulcus. Moreover, as the swing parameters of the wave electric toothbrush increase, the gingival sulcus cleaning efficacy improves. With higher brush head swing parameters, the larger angle of oscillation allows the bristles to penetrate deeper into the gingival sulcus, with the reciprocating movement of the brush head causing the bristles to repeatedly rub against the sulcus. For example, at tooth position 16, it is evident that the maximum gingival sulcus cleaning depth increases from 1.39 ± 0.23 mm to 2.60 ± 0.36 mm as the swing parameters of the wave electric toothbrush progress from low to high. Therefore, the second null hypothesis is rejected. Furthermore, both preclinical and clinical studies have confirmed that the deposition and attachment of biofilm on the implant-prosthesis structure are the primary factors leading to the onset and persistence of peri-implant inflammation. Thus, the wave electric toothbrush, with its ability to deeply clean the gingival sulcus, will be used to help prevent peri-implant inflammation and contribute to the long-term success of implants [43,44].

Previous studies have shown that both electric toothbrushes and manual brushing may have an impact on soft tissues. During tooth brushing, the superficial keratinized layer of the gingival tissue may be removed, exposing the underlying connective tissue, leading to gingival abrasion. Additionally, soft tissue damage may be influenced by multiple factors, such as bristle hardness [45,46]. In terms of soft tissue safety, brushing the first to third molars in the upper molar region of SD rats using the wave electric toothbrush with different swing parameters resulted in varying levels of soft tissue bleeding. Therefore, the third null hypothesis is rejected. However, the number of oral bleeding episodes caused by the wave electric toothbrush in SD rats was relatively low and occurred only in cases with medium and high swing parameters. Over the 30-day brushing experiment, the number of bleeding episodes ranged from 1.2 ± 1.1 to 3.3 ± 2.8. The manual Bass technique involves inserting the bristles at a 45° angle into the gingival sulcus and performing horizontal vibrations at 1.4 times per second, while the wave electric toothbrush oscillates reciprocally at high frequencies of approximately 2.2 times per second for low swing parameters (brush head swing angle: 9°), 2.4 times per second for medium swing parameters (brush head swing angle: 14°), and 3.2 times per second for high swing parameters (brush head swing angle: 22°). Therefore, the bristles of the wave electric toothbrush head are more likely and more frequently to come into contact with the soft tissues around the buccal and lingual-palatal regions of the teeth, thereby scraping the epithelial cells. Moreover, the oral mucosal epithelium is thin, and the connective tissue attachment at the gingival margin and gingival sulcus is loose, making it more prone to being lifted or detached by external forces [47]. As such, under low swing parameters, frequent brushing does not cause severe soft tissue damage. In contrast, larger oscillations of the brush head increase the contact area between the bristles and soft tissues, making it more likely to cause damage, particularly in areas like the vestibular sulcus in SD rats. In addition, the anatomical map of the SD rats’ oral cavity confirmed that significant bleeding sites were observed in the oral vestibule and palatal gingiva. These findings suggest that larger oscillations of the wave electric toothbrush brush head increase the likelihood of soft tissue damage and bleeding in the oral cavity of SD rats.

By observing the structure and cell morphology of oral soft tissues, the balance between the mechanical forces exerted during tooth brushing with the wave electric toothbrush and the soft tissue tolerance was analyzed. Soft tissue cells from areas of the SD rat oral cavity most susceptible to bristle friction were selected for H&E staining. The results showed that brushing with different techniques induced varying changes in the oral soft tissue structure of the SD rats. Moreover, the degree of tissue damage caused by the wave electric toothbrush with different swing parameters varied. Similarly to the results of manual Bass brushing, the wave electric toothbrush with low swing parameters caused localized thinning of the epithelium, with a few epithelial cells showing vacuolar degeneration and a small number of inflammatory cells. However, as the swing parameters of the wave electric toothbrush increased, the extent of epithelial damage progressed from small areas of erosion and collagen fiber rupture, with an increase in inflammatory cells, to a state where full-thickness epithelial rupture occurred, exposing the underlying connective tissue. This resulted in epithelial cell damage, disordered cell arrangement, and an increased density of inflammatory cell infiltration. And these results are consistent with previous studies, which suggest that moderate mechanical stimulation can promote the secretion of anti-inflammatory factors, while excessive mechanical stimulation may lead to soft tissue inflammation [48]. Therefore, after epithelial layer damage, exposure of the basement membrane triggers the activation of local immune cells, leading to the release of inflammatory cytokines and initiating an inflammatory response [49]. The mechanical friction generated by the bristles of the wave electric toothbrush during high-frequency oscillation induces external damage signals to the oral soft tissue cells in rats, such as epithelial cells, fibroblasts, and macrophages. These cells activate the inflammatory factor secretion through their stress response system [50]. Among them, the NF-κB pathway is one of the core pathways regulating the gene expression of inflammatory factors. It is activated by the mechanical stimulation from the brush head, triggering the expression of TNF-α and IL-1β [51,52]. Additionally, the expression levels of TNF-α and IL-1β serve as direct quantitative indicators of the intensity of the inflammatory response [53]. Therefore, during the toothbrushing process, the high-frequency oscillation of the bristles driven by the brush head causes damage to the oral soft tissue cells in SD rats, leading to an inflammatory response. And the degree of tissue and cell damage, as well as the intensity of the inflammatory response, are highly correlated with the swing parameters of the wave electric toothbrush.

Overall, the results of this study indicate that the wave electric toothbrush offers effective oral cleaning, particularly in the cleaning of the gingival sulcus. Specifically, the wave electric toothbrush demonstrated comprehensive cleaning of buccal surface plaque on a human oral model across low, medium, and high swing parameters, with the cleaning range of gingival sulcus plaque increasing as the swing parameters were enhanced. Therefore, it presents a potential alternative to the manual Bass technique. Nevertheless, it is important to note that these results were obtained when the wave electric toothbrush was used by trained dental professionals following scientifically established methods. Therefore, before applying the experimental results obtained in this study to daily practice, it is essential to fully understand the usage methods and cleaning principles of the wave electric toothbrush. While this study systematically evaluated the subgingival plaque cleaning efficacy and safety of the wave electric toothbrush, several limitations should be acknowledged. First, the focus on periodontal health led to the adoption of the Bass technique, emphasizing sulcular cleaning; thus, its efficacy in caries prevention (e.g., on occlusal surfaces) was not thoroughly examined. Second, our findings are derived from healthy animal models and lack validation in human populations, particularly those with active periodontal disease. Third, the study assessed a limited set of parameters, without establishing comprehensive time-course or quantitative parameter-effect relationships. Finally, the research focused on histological outcomes and did not explore underlying cellular mechanisms (e.g., inflammatory signaling). However, as pointed out in recent research about conventional powered and experimental toothbrushes, the overall scarcity of clinical trials on this topic precludes definitive conclusions regarding the superiority of any specific toothbrush design over another [17,54]. To address these gaps, future research should: 1. conduct long-term clinical trials in diverse human populations to validate efficacy and establish guidelines; 2. perform systematic parameter-optimization studies to define the quantitative relationships between key brushing parameters and clinical outcomes; 3. utilize molecular techniques to elucidate cellular mechanisms; and 4. evaluate the brush’s effectiveness from a cariology perspective to provide a comprehensive public health assessment. 5. conduct clinical trials comparing the wave electric toothbrush against leading powered toothbrush technologies to establish its relative efficacy and safety in human populations.

## 5. Conclusions

This study evaluated the subgingival cleaning efficacy and oral soft tissue safety of the wave electric toothbrush through in vitro and in vivo experiments. The results demonstrated that the wave electric toothbrush provides effective subgingival cleaning, with its efficacy improving as the swing parameters of the brush head increase. Furthermore, oral soft tissue safety was closely associated with the swing parameters of the toothbrush, where higher parameters correlated with an increased risk of soft tissue damage.

## Figures and Tables

**Table 1:** Mean values and standard deviations of buccal surface cleaning rate (BSCR), gingival sulcus cleaning rate (GSCR), and maximum gingival sulcus cleaning depth (MGSCD) for different tooth positions (TP) obtained from the in vitro cleaning experiment.

Group	TP	BSCR (%)	GSCR (%)	MGSCD (mm)
Control (*n* = 6)	16	0	0	0
	26	0	0	0
	36	0	0	0
	46	0	0	0
Manual (*n* = 6)	16	100	44.1 ± 1.9	1.32 ± 0.14
	26	100	28.7 ± 3.5	1.39 ± 0.07
	36	100	38.2 ± 7.7	1.62 ± 0.47
	46	100	42.6 ± 2.2	1.31 ± 0.36
Low (*n* = 6)	16	100	43.4 ± 2	1.39 ± 0.23
	26	100	28.6 ± 3.7	1.55 ± 0.31
	36	100	38.1 ± 1.1	1.85 ± 0.76
	46	100	35.7 ± 1.7	1.50 ± 0.29
Medium (*n* = 6)	16	100	58.8 ± 6.7	1.87 ± 0.29
	26	100	49.1 ± 1.3	2.00 ± 0.08
	36	100	52.4 ± 7.5	2.30 ± 0.10
	46	100	52.7 ± 5.3	2.02 ± 0.52
Large (*n* = 6)	16	100	68 ± 15.1	2.60 ± 0.36
	26	100	54.7 ± 0.4	2.76 ± 0.25
	36	100	62.5 ± 11.5	2.28 ± 0.22
	46	100	55.7 ± 9.7	2.13 ± 0.13

**Table 2:** Number of soft tissue bleeding episodes during toothbrushing in SD rats.

Group	Control	Manual	Low	Medium	Large
Bleeding Frequency	0	0	0	1.2 ± 1.1	3.3 ± 2.8

## Data Availability

Available under request to the corresponding author.
